# Assorted Processing of Synthetic Trans-Acting siRNAs and Its Activity in Antiviral Resistance

**DOI:** 10.1371/journal.pone.0132281

**Published:** 2015-07-06

**Authors:** Mingmin Zhao, David San León, Frida Mesel, Juan Antonio García, Carmen Simón-Mateo

**Affiliations:** 1 Departamento de Genética Molecular de Plantas, Centro Nacional de Biotecnología (CNB-CSIC), Campus Universidad Autónoma de Madrid, 28049, Madrid, Spain; 2 College of Agriculture, Yangtze University, Jingzhou, Hubei, 434025, P.R. China; University of California, Riverside, UNITED STATES

## Abstract

The use of syn-tasiRNAs has been proposed as an RNA interference technique alternative to those previously described: hairpin based, virus induced gene silencing or artificial miRNAs. In this study we engineered the TAS1c locus to impair *Plum pox virus* (PPV) infection by replacing the five native siRNAs with two 210-bp fragments from the CP and the 3´NCR regions of the PPV genome. Deep sequencing analysis of the small RNA species produced by both constructs *in planta* has shown that phased processing of the syn-tasiRNAs is construct-specific. While in syn-tasiR-CP construct the processing was as predicted 21-nt phased in register with miR173-guided cleavage, the processing of syn-tasiR-3NCR is far from what was expected. A 22-nt species from the miR173-guided cleavage was a guide of two series of phased small RNAs, one of them in an exact 21-nt register, and the other one in a mixed of 21-/22-nt frame. In addition, both constructs produced abundant PPV-derived small RNAs in the absence of miR173 as a consequence of a strong sense post-transcriptional gene silencing induction. The antiviral effect of both constructs was also evaluated in the presence or absence of miR173 and showed that the impairment of PPV infection was not significantly higher when miR173 was present. The results show that syn-tasiRNAs processing depends on construct-specific factors that should be further studied before the so-called MIGS (miRNA-induced gene silencing) technology can be used reliably.

## Introduction

The term RNA silencing describes an ensemble of regulatory pathways that share the key role played by diverse sets of endogenous small RNAs (sRNAs) of 21–24 nt in length [1]. These sRNAs are initially produced by Dicer-like (DCL) endonucleases that process double-stranded RNA precursors [2]. Two specialized types of sRNAs are produced in plants: microRNAs (miRNAs), which are excised from partially double-stranded regions of hairpin structures, and small interfering RNAs (siRNAs), which are processed from perfectly complementary double-stranded molecules [3]. One strand of each miRNA or siRNA duplex is loaded into effector protein complexes including a protein of the Argonaute (AGO) family, and guide them to either degradation or translation suppression of target RNAs or to chromatin rearrangements at specific places [4–6].

In plants, primary products of some miRNA- and siRNA-directed cleavages serve as templates for RNA-dependent RNA polymerases (RDR), to generate double stranded RNA, which is processed into a second wave of siRNAs [7–9]. These so-called secondary siRNAs amplify and reinforce RNA silencing, and virus-derived secondary siRNAs have been shown to play a main role in antiviral immunity [10]. Several factors have been described that contribute to define which loci generate secondary siRNAs: i) aberrant ends of the targeted RNA [11], ii) proximity of two sRNA target sites [12], iii) targeting by sRNAs loaded in particular AGO proteins [13,14], iv) appropriate sRNA structure (22-nt size, bulged sRNA duplex) [13,15,16].

Some secondary siRNAs are 21-nt phased (phased siRNAs) as a result of successive DCL-catalyzed processings from the end of a dsRNA substrate originated by an RDR from an AGO-catalyzed cleaved RNA at a miRNA target site [2,17]. Phased siRNAs that are able to direct repression at loci different from that the ones they derive, are termed *trans*-acting siRNAs (tasiRNAs) [2]. TasiRNAs are very abundant in some plant families as *Solanaceae* and *Fabaceae*, but they are not well conserved between different plant species [18]. TasiRNAs play key regulatory roles in plant development [19,20] and have been proposed to coordinate the repression of large gene families as that of *Pentatricopeptide Repeat* (*PPR*) genes [21,22] or the nucleotide-binding site-leucine-rich repeat (NBS-LRR) family of resistance genes [23–26].

Four families of genes coding for tasiRNA precursors (*TAS*), comprising eight different loci have been identified in the *Arabidopsis thaliana* genome [18]. Whereas *TAS*3 family, which generates tasiRNAs by the two-hit mechanism triggered by miR390 loaded in the specialized argonaute AGO7, is widely conserved in moss and higher plants, genes of *TAS1/TAS2* families, whose primary transcripts are targeted by a single hit of the 22-nt-long version of miR173, are unique to *Arabidopsis* and closely related species [12,27].

MiR173-triggered production of tasiRNAs can be settled in heterologous plants, such as *Nicotiana benthamiana*, and has been used to engineer single or multiple copies of synthetic tasiRNAs (syn-tasiRNAs) able to silence endogenous genes, including *FAD2* [28], *PDS* [29], *CH42* [30] and *FT* or *TRY/CPC/ETC2* [31]. This gene silencing technology, recently dubbed as miRNA-induced gene silencing (MIGS), can reliably knockdown single genes or multiple unrelated genes [32].

Artificial miRNAs (amiRNAs) has been shown as a valuable alternative to RNA silencing approaches based on the expression of large virus-derived sequences to generate antiviral resistance [33–35]. In this study we wanted to gain insight into general and particular features of syn-tasiRNAs generated by MIGS and to explore the antiviral potential of this technology, using as experimental system the infection of *Plum pox virus* (PPV), a positive strand RNA virus of the genus *Potyvirus* [36]. We engineered the *TAS1c* locus to produce syn-tasiRNAs targeting either the 3’ noncoding region (NCR) or the capsid protein (CP) coding region of PPV RNA. We used a transient agro-infiltration assay in *Nicotiana benthamiana* and Illumina deep-sequencing analysis, to assess the small RNA accumulation and the susceptibility to PPV infection of plants expressing the PPV-targeted syn-tasiRNA constructs alone or co-expressed with *A*. *thaliana* miR173 precursor.

## Results

### PPV-specific synthetic tasiRNA (syn-tasiRNA) constructs

**Fig 1:**
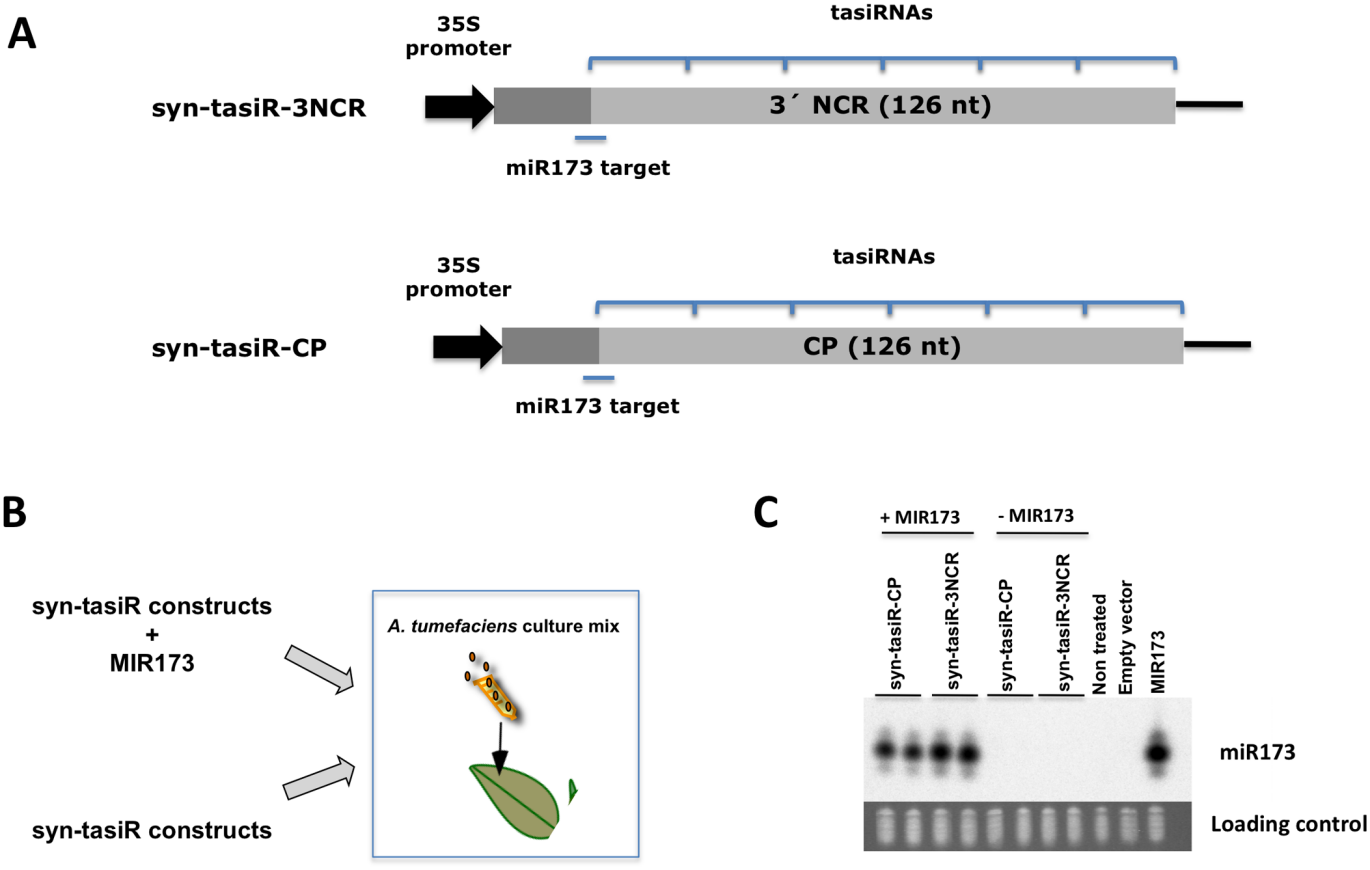
Syn-tasiRNA constructs and miR173 accumulation in *N*. *benthamiana* transient assays. **A)** Diagram of TAS1-derived syn-tasi constructs containing PPV sequences. The tasiRNA-spawning region is indicated by brackets and the length of the PPV regions included in the constructs is shown. The miR173 target site is indicated by a line. **B)** Agroinfiltration transient assay in *N*. *benthamiana*. Syn-tasiRNA constructs were expressed individually or in combination with miR173 precursor. **C)** Blot assay to assess the accumulation of miR173 in the plant tissue three days after agroinfiltration. Two biological replicates are shown. EtBr-stained 5S rRNA/tRNA is shown.

TAS1-like constructs designed to produce siRNAs targeting the different regions of the PPV genome were engineered. We generated two constructs that included the miR173 target site (22 nt) and two upstream nucleotides of TAS1a and c, immediately followed by 126 nt from either the 3’NCR (nt 9529–9654) or the CP coding region (nt 9242–9367) of PPV. These constructs were cloned downstream a 35S promotor into pMDC32 yielding syn-tasiR-3NCR and syn-tasiR-CP (Fig 1A). In order to produce in *N*. *benthamiana* the miR173 required to trigger syn-tasiRNA formation from syn-tasiR-3NCR and syn-tasiR-CP, we generated a construct, MIR173, containing the AtmiR173 precursor (509 nt) [29]. The combined expression of either syn-tasiR-3NCR or syn-tasiR-CP and MIR173 is predicted to yield up to six phased syn-tasiRNAs designed to target PPV RNA.

### miR173 expression increases 21-nt sRNA accumulation from syn-tasiRNA constructs downstream the miR173 target site

Previous studies about synthetic tasiRNAs have mainly focussed on their functional activity. In order to gain deeper information on how a miR173 target site contributes to downstream production of phased siRNAs, we checked by high throughput sequencing the production of small RNAs from syn-tasiR-3NCR or syn-tasiR-CP transiently expressed by agroinfiltration in *N*. *benthamiana* leaves. *Agrobacterium tumefaciens* cells bearing either of these constructs were co-infiltrated with cells expressing MIR173 (Fig 1B) (for simplicity, we will refer to the *A*. *tumefaciens* bacteria by the plasmid they carry). As a control, syn-tasiRNA constructs were also infiltrated without the miR173-expressing plasmid. At three days post-agroinfiltration (3 dpa), small RNAs (sRNAs) were extracted from the infiltrated leaves. sRNA samples from leaves expressing syn-tasiR-3NCR and syn-tasiR-CP were pooled and subjected to deep sequencing analysis. Northern blot analysis verified that miR173 accumulated at detectable levels only in the *N*. *benthamiana* leaves infiltrated with MIR173 (Fig 1C).

**Fig 2:**
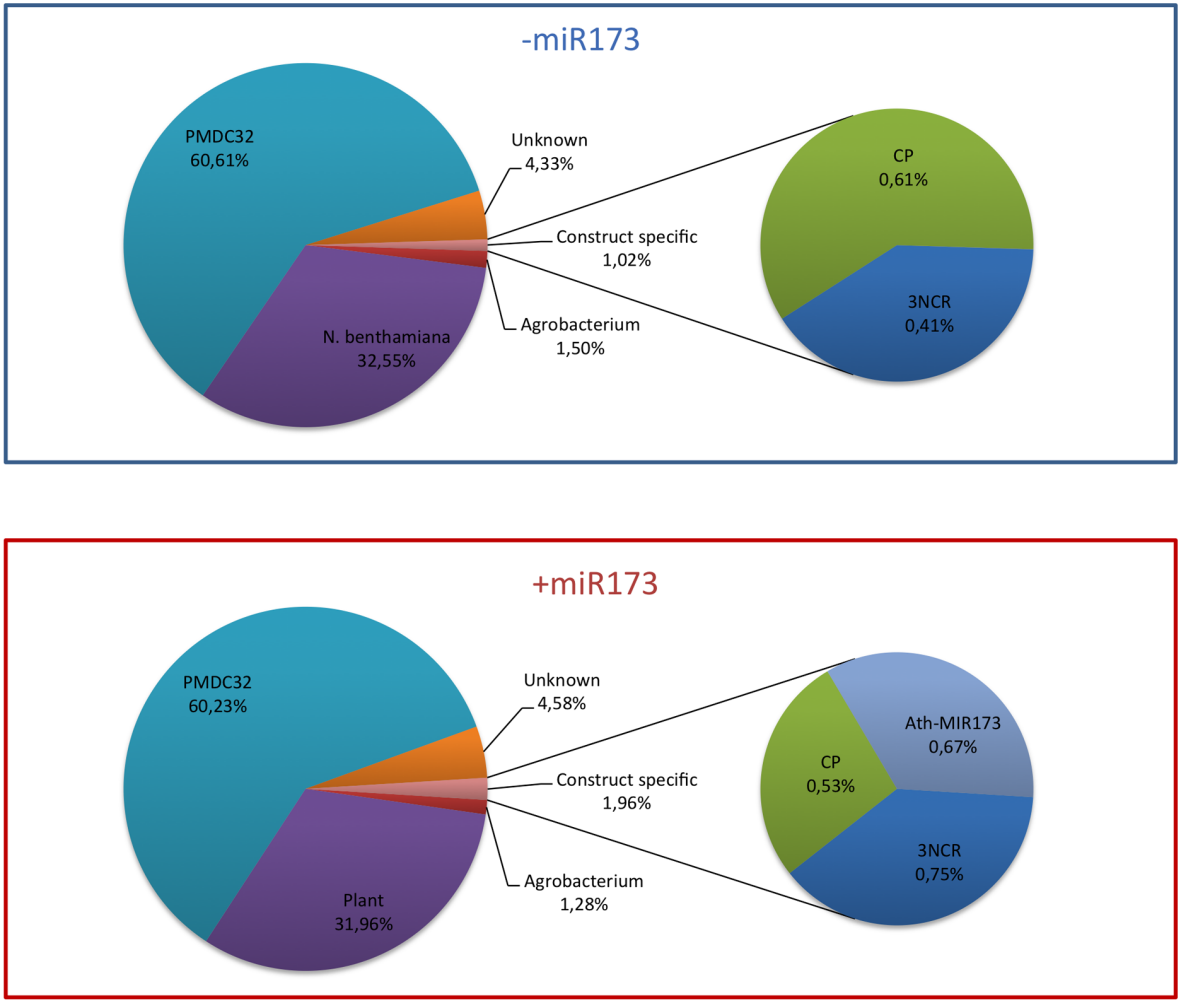
Effect of miR173 expression for syn-tasiRNA accumulation in *N*. *benthamiana* transient assays. Mapping of small RNA sequences obtained by high-throughput sequencing are shown. Analyses of total small RNA (18–26 nt) reads in the presence (+miR173) or absence of miR173 (-miR173) is shown in percentages in pie charts. Those reads mapping across the syn-tasiRNA constructs (construct specific) are shown in detail. CP: reads mapping to CP region included in the syn-tasiR-CP. 3NCR: reads mapping to 3’NCR region included in the syn-tasiR-3NCR. Ath-MIR173: reads mapping to the MIR173 construct.

The total numbers of reads after filtering to remove rRNA and tRNA were 9,433,876 and 8,893,736 for the pools of syn-tasiRNA constructs expressed alone (library-miR173) or together with miR173 (library +miR173), respectively. To determine how expression of miR173 affects the patterns of sRNA populations, we classified sRNAs in five groups: i) matching to *N*. *benthamiana* genome, ii) matching *Agrobacterium* genome, iii) matching common regions of agroinfiltrated pMDC32-derived plasmids, iv) matching specific regions of each pMDC32-derived plasmids, and v) unknown sequences (Fig 2). Approximately 32%, 1.5% and 60% of the reads of the-miR173 library corresponded to sRNAs of the *N*. *benthamiana*, *Agrobacterium* and pMDC-shared groups, respectively (Fig 2). These percentages were marginally lower, and that of the unknown sequences marginally higher, in the +miR173 library (Fig 2). As corresponds to the small size of the cloned PPV sequence, the percentages of both PPV CP- and PPV 3’NCR-specific reads were low in both libraries. Interestingly, the percentage of 3’NCR-specific reads was 83% higher in the library derived from leaves expressing miR173 (0.75% versus 0,41%) (Fig 2), suggesting that miR173 enhanced the production of siRNAs downstream of its target site. However, an equivalent increase of CP-specific siRNAs as consequence of miR173 expression was not observed (Fig 2).

**Fig 3:**
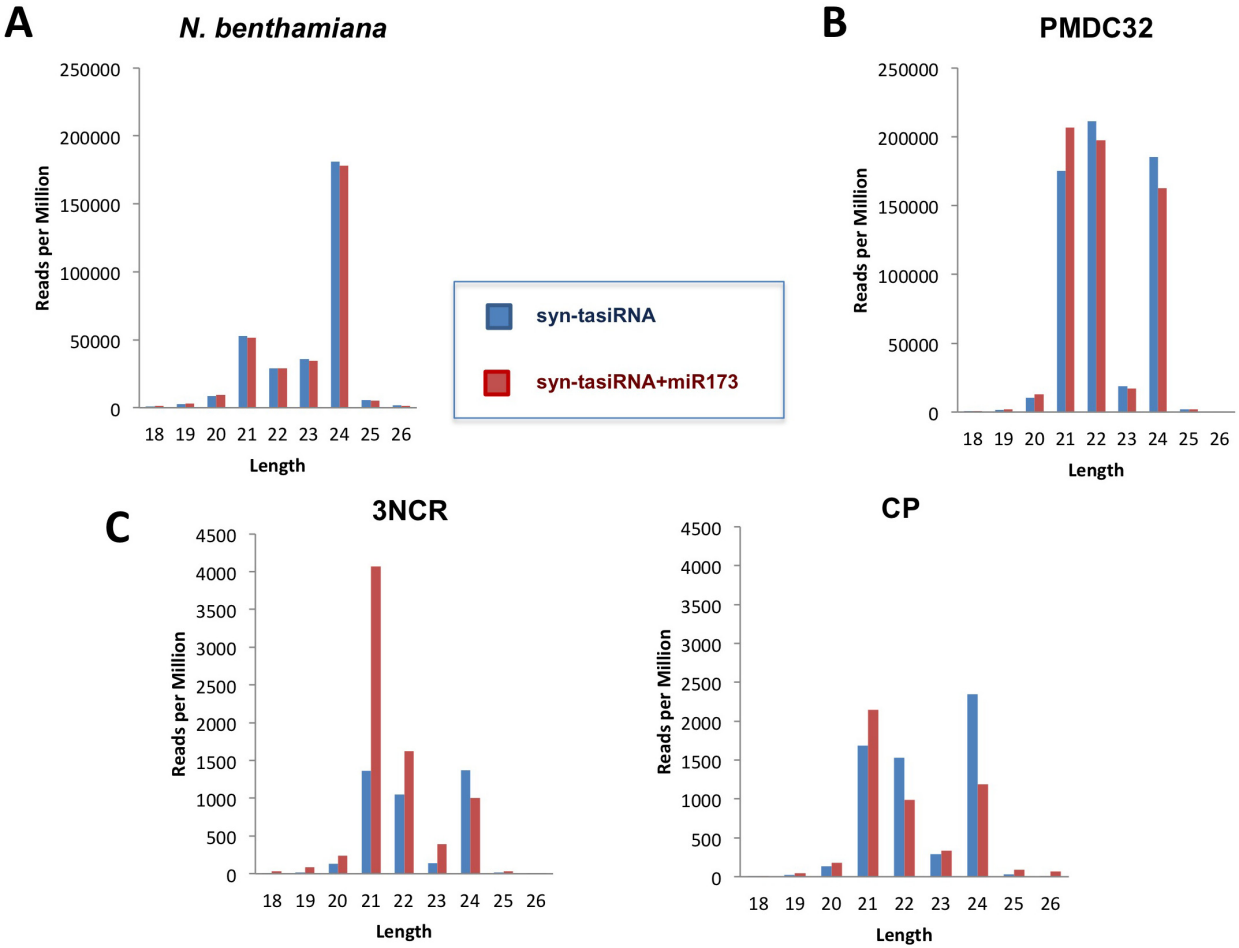
Effect of miR173 expression in size distribution profiles of syn-tasiRNAs produced in *N*. *benthamiana* transient assays. Size distribution of small RNAs (18–26 nt) identified by high-throughput sequencing is shown. **A)** Small RNAs mapping to *N*. *benthamiana* genome. **B)** Small RNAs mapping to the common region of pMDC32-derived plasmids. **C)** Small RNAs mapping to PPV-derived regions from syn-tasiR-3NCR (3NCR) and syn-tasiR-CP (CP) constructs.

The size distribution profiles of the-miR173 and +miR173 sRNA libraries (Fig 3) confirmed previous reports that 24-nt is the major species of endogenous sRNAs of *N*. *benthamiana* [37] and showed that expression of miR173 had no significant effect in the size distribution of endogenous populations of sRNAs (Fig 3A). In contrast, the size distribution profile of exogenous sRNAs, derived from pMDC-based plasmids showed peaks of similar height at 21 nt and 24 nt (Fig 3B and 3C). Moreover, expression of miR173 did not significantly alter the size distribution of sRNAs derived from pMDC-shared regions (Fig 3B). With respect to the size distribution of the sRNAs from PPV specific regions the pattern was very similar to those of pMDC-shared regions in the absence of miR173 (Fig 3C). However miR173 expression caused a large increase in the proportion of PPV 3’NCR-specific 21-nt sRNAs, which was much less pronounced for PPV CP-specific siRNAs. These data suggest that cleavage at a miR173 target site enhances the accumulation of 21-nt sRNAs derived from downstream sequences in the syn-tasiRNA constructs.

### miR173 expression induces the production of phased 21-nt siRNAs from syn-tasiRNA constructs downstream the miR173 target site

In order to determine particular features of sRNAs whose accumulation is enhanced as a consequence of miR173 expression, thus discarding sRNAs derived from non-specific induction of PTGS, we identified sRNA species that were abundant in the +miR173 sample (more than 50 reads) and accumulated in larger amounts as compared to sRNAs originated in the absence of miR173 (at least 8 times more). A very low amount of species fulfilling these thresholds were detected among the *N*. *benthamiana*, *Agrobacterium* or unknown sRNAs and among sRNAs mapping to the common region of pMDC32- derived plasmids. In contrast, 34 and 22 sRNAs species derived from sequences downstream the miR173 target sites of syn-tasiR-CP and syn-tasiR-3NCR, respectively, met the threshold and increased when miR173 was expressed (S1 Table).

**Fig 4:**
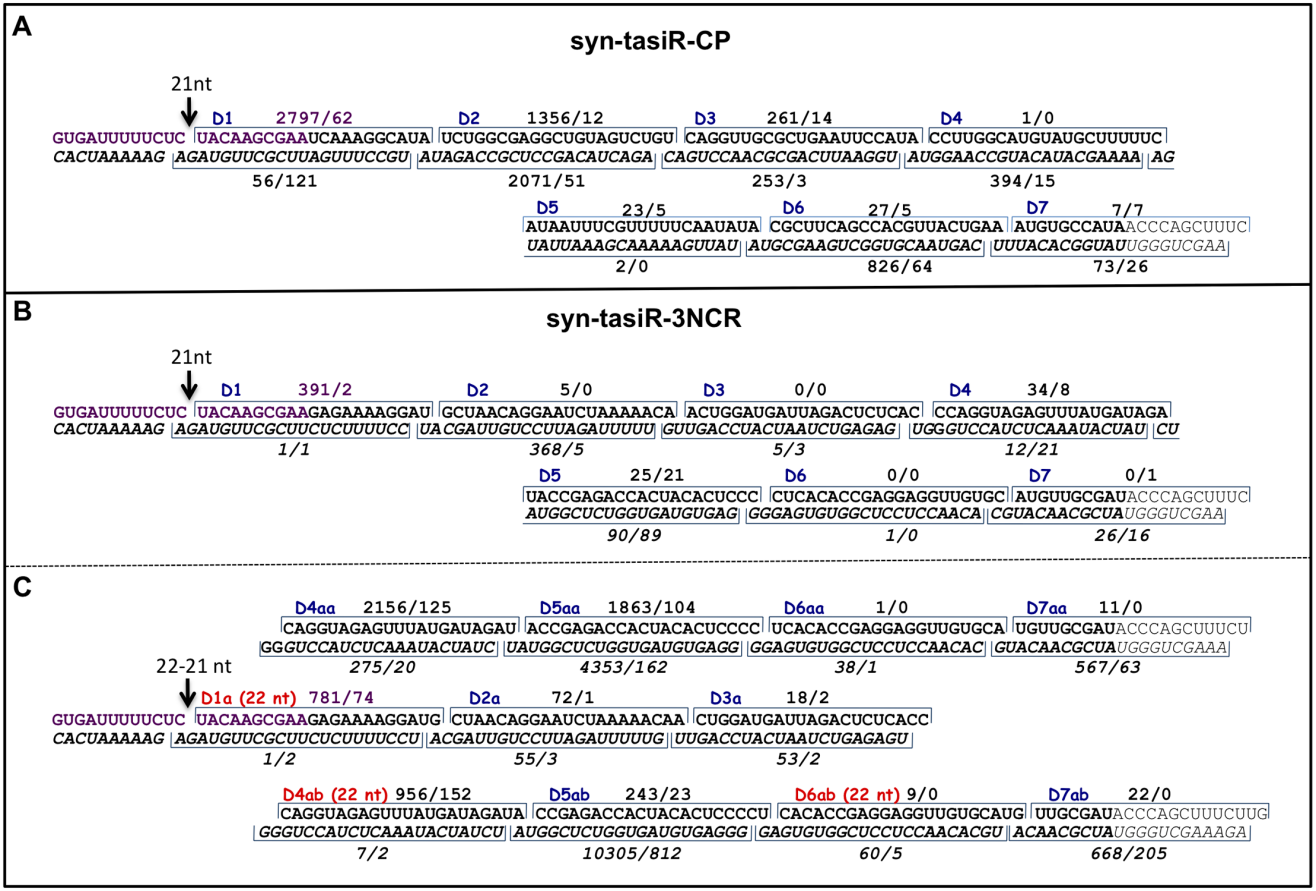
In-phase processing of syn-tasiRNA precursors directed by miR173. Phased syn-tasiRNAs (21 nt) (D1-D7) in register with the miR173-guided cleavage site (indicated with an arrow) from syn-tasiR-CP **(A)** and syn-tasiR-3NCR **(B)** constructs. **(C)** Two series of phased syn-tasiRNAs (21 and 22 nt) in register with the miR173-guided cleavage site (indicated with an arrow). One series is marked as a-aa and the second one as a-ab. Numbers above and below the syn-tasiRNAs indicate reads from samples syn-tasiR+173/syn-tasiR-173. The mi173 target site (in purple) and the PPV sequences are shown in bold.

Seven PPV CP-specific sRNAs induced by miR173-expression were 21-nt in length and were in register with the expected miR173-guided cleavage site of the syn-tasiR-CP transcript (Fig 4A). In agreement with its origin from double stranded products, some of these sRNAs mapped to the transcribed strand [D1(+), D2(+) and D3(+)] and some others did to the complementary strand [D2(-), D3(-), D4(-) and D6(-)]. Whereas similar accumulation of both strands was observed for some of the PPV CP-specific phased sRNAs (D2 and D3), in other cases, one strand, either the positive (D1), or the negative (D4 and D6), was strongly preferred (Fig 4A). In the case of the syn-tasiR-3NCR two contiguous 21-nt sRNAs [D1(+) and D2(-)] laying just downstream the miR173 cleavage site were shown to be induced in response to miR173 (Fig 4B) but the 21-nt register did not go beyond. However, eight phased miR173-induced 21-nt sRNAs that accumulated more than 50 reads in the +miR173 library could be coupled to the expected miR173-guided cleavage site by a 22-nt sRNA [D1a(+)], which was also induced when miR173 was expressed (Fig 4C, species a and aa). But this was not the only register of phased sRNAs. Two other 22-nt sRNAs species [D4ab(+) and D6ab(-)] allow to couple another series of phased sRNAs, which included nine species fulfilling the threshold conditions when miR173 is expressed (Fig 4C, species a and ab); one of them, D5ab(-), accumulates up to 10305 reads.

**Fig 5:**
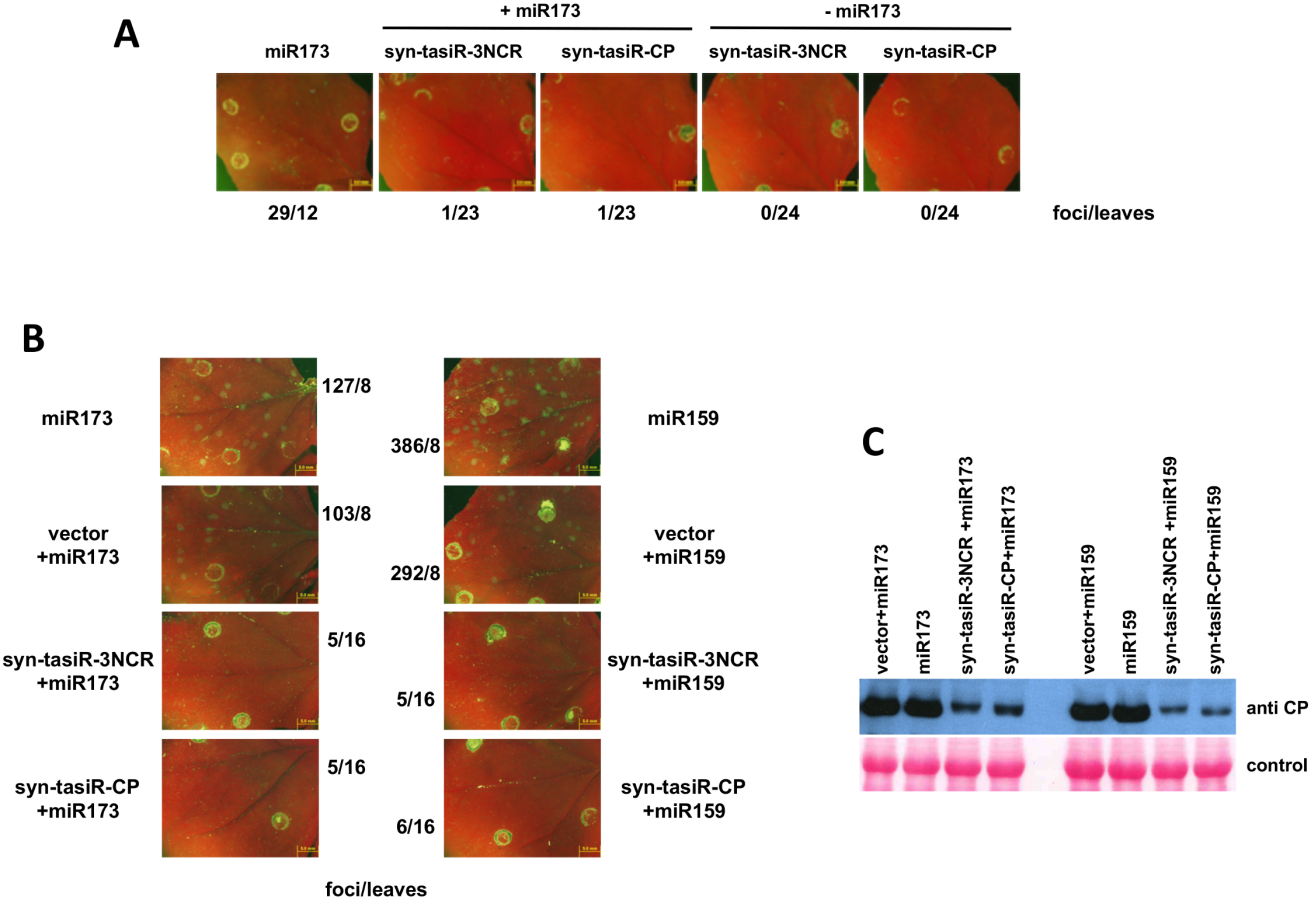
Antiviral effect of PPV-specific syn-tasiRNA constructs. **(A** and **B**) The indicated combinations of MIR173 (miR173), MIR159 (miR159), empty pMDC32 (vector) syn-tasiR-3NCR and syn-tasiR-CP were transiently expressed by agroinfiltration in *N*. *benthamiana* plants. Three days post-agroinfiltration plants were inoculated with PPV-GFP and 5 days post-inoculation (dpi) infection foci were observed under a fluorescence microscope. Number of foci/leaves is indicated. **(C)** Agroinfiltrated leaves harvested at 5 dpi were subjected to immunoblot analysis with anti-CP serum. The membrane stained with Ponceau red is included as loading control.

The results presented above clearly show that the sRNAs accumulation patterns observed upon expression of syn-tasiR-CP and syn-tasiR-3NCR constructs are significantly different. The sRNAs species observed from the processing of syn-tasiR-CP construct fulfill with the expected ones, that is, seven 21-nt phased sRNAs in register with the predicted miR173-guided cleavage site (Fig 4A). However, this accumulation pattern is not the one favored in the processing of syn-tasiR-3NCR constructs (Fig 5B). Two different patterns can be envisaged in which a 22nt sRNA specie [D1a(+)] is the first observed in register with the predicted miR173-guided cleavage site (Fig 4C). In one pattern, 21-nt sRNAs (species a and aa) in phase with D1a accumulate while in a second pattern D1a species are followed by phased sRNAs (species a and ab) in which species of 21-nt and 22-nt alternate (Fig 4C). These results highlight that, although production of phased siRNAs in register with a miR173 cleavage happens, specific features of the phased series might depend on each particular downstream sequence.

### syn-tasiR-3NCR and syn-tasiR-CP expression protect *N*. *benthamiana* plants against PPV infection

To test the antiviral activity of syn-tasiR-3NCR and syn-tasiR-CP, we design an experimental approach in which the PPV-specific syn-tasiRNA constructs were delivered to *N*. *benthamiana* by agroinfiltration and the infiltrated leaves were challenged three days later by mechanical inoculation with purified virions of a recombinant PPV expressing GFP. Whereas an average of 2.4 infection foci per leaf were detected on the plants expressing only miR173, plants expressing syn-tasiR-3NCR or syn-tasiR-CP, either with or without miR173, showed high resistance to PPV: a single focus was detected on one of the 23 leaves treated with syn-tasiR-3NCR or syn-tasiR-CP plus miR173 and no focus was detected on any of the 24 leaves expressing syn-tasiR-3NCR or syn-tasiR-CP alone (Fig 5A).

To assess the strength of the resistance, agroinfiltrated plants were challenged with a higher virus dose (450 ng/leaf). The number of infection foci of plants expressing syn-tasiR-3NCR or syn-tasiR-CP, either plus miR173 or plus miR159 as a control, was drastically reduced, compared with plants agroinfiltrated with plasmids expressing miR173 or miR159, either alone or together with an empty vector (5 or 6 foci in 16 leaves in comparison with between 103 and 386 foci in 8 leaves) (Fig 5B). Immunodetection of PPV CP by a Western assay confirmed the antiviral activity of syn-tasiR-3NCR and syn-tasiR-CP, which was not enhanced by co-expression of miR173 (Fig 5C).

## Discussion

RNA silencing is a natural regulatory and defense mechanism that has been engineered as a useful biotechnological tool to downregulate plant gene expression and to provide antiviral resistance [1,38]. MIGS technology, based on syn-tasiRNAs [32], has been recently added to the repertoire of RNA silencing-related strategies to suppress gene expression, which also includes more classical approaches based on sense or inverted repeat RNAs [39,40], and amiRNAs [34,41,42]. In this paper we present evidence showing that phased processing of pre-syn-tasi RNAs can be more complex and construct-specific that previously speculated, and that syn-tasi-RNA technology might be not very advantageous when compared with other RNA silencing-based approaches.

We have used the previously reported TAS1/miR173-related system [29,32,43] to express PPV-specific tasiRNAs in *N*. *benthamiana*. The two syn-tasiRNA constructs used in this study only differ in 126 nt which correspond to two different regions (CP coding sequence and 3’ NCR) of the PPV genome, placed downstream of a miR173 cleavage site. PTGS induction by these constructs was evidenced by accumulation of a large number of sRNAs spread through the complete T-DNA region, regardless coexpression of miR173. We also detected sRNAs specifically induced by miR173 expression which mapped almost exclusively downstream the miR173 target site, which is in agreement with previous reports showing that TAS1-like transcripts yield tasiRNA only from the 3’ RNA fragment generated by miR173-guided cleavage [17,29]. Abundant sRNAs derived from the syn-tasiR-CP construct were in phase following a 21-nt register, as it has been previously shown for other tasi- and syn-tasi-RNAs [29,31,44]. A similar series of phased sRNAs was not detected for syn-tasiR-3NCR. In contrast, a 22-nt species collinear with this sRNA served as leader of two series of phased small RNAs, one of them in an exact 21-nt register, and the other one in a mixed 21-/22-nt frame. This is not the first observation of phased tasiRNAs that do not follow a strict 21-nt register, since processing offset by 1nt after the forth cleavage cycle was reported in the Arabidopsis *TAS1c* transcript [29], although the species involved in this register shift were not mentioned. Tasi-RNAs are thought to derive from DCL4-cleavage of RNA precursors synthesized by RDR6 [21, 44]. Since DCL4 cleavage produces 21-nt sRNAs, the detection of miR173-induced 22-nt sRNAs in the phased register of some tasiRNAs, supports previous results suggesting that DCL2 might be also involved in the processing of tasiRNA-precursors [45, 46]. In any case, our results demonstrate that, whereas cleavage by miR173 consistently gives rise to the production of double stranded RNA that is further processed in phased sRNAs, the exact register of cleavage depends on specific features of the RNA placed downstream the miR173 target. These features, probably related with specific recognition by different DCL species, remain to be identified.

Montgomery et al. [29] showed that 78% of the 21-nt phased sRNAs derived from a GFP-related syn-tasiRNA placed downstream a miR173 target site corresponded to the second-phase cycle (plus and minus polarities were computed together). Similarly, D2(+) and D2(-), together with the sRNA adjacent to the miR173 cleavage site, were the most abundant phased species derived from syn-tasiR-CP. However, species at position D2 of syn-tasiR-3NCR were rare, and minus strand of the fifth sRNAs of the second phased series (species aa and ab) showed by far the largest numbers of reads. Whereas D1(+) and D2(+) species of syn-tasiR-CP have a 5’ terminal U, the 5’ terminal residue of syn-tasiR-CP D2(-) is A and those of syn-tasiR-3NCR D5aa(-) and D5ab(-) are G. The conclusions about sRNA abundance deduced from deep sequencing reads, have to be taken with caution, since it has been shown that deep sequencing can produce biased quantitative results [47]. But, with these reservations, our data seem to indicate that neither the position relative to the miR173 cleavage site, nor the strand polarity, nor the 5’ terminal residue are decisive factors to define the sRNA abundance. Both the 5’ terminal nucleotide [48] and the relative stabilities of the base pairs at the 5’ ends of the sRNA duplex [49, 50] determine the efficiency of loading in RISC and, thus, survival of each sRNA strand. However, we have not found correlation between particular 5’ terminal nucleotides or specific free-energy bias at the sRNA ends and miR173-induced sRNA accumulation.

The abundance of PPV-derived sRNAs in leaves agroinfiltrated with syn-tasiR-CP and syn-tasiR-3NCR in the absence of miR173 indicates that these constructs are inducing a strong sense PTGS reaction. In agreement with this fact, these agroinfiltrated leaves showed an efficient anti-PPV protection, which was not significantly enhanced by tasiRNA production induced by miR173 expression. Recently, Carbonell et al. [31] also showed that syn-tasiRNAs did not cause RNA silencing more efficiently that traditional amiRNAs. Thus, whereas syn-tasiRNAs are efficient tools to engineer silencing of gene expression, they do not appear to offer a substantial enhancement of efficiency compared to former RNA silencing strategies. Our results highlight the existence of construct-specific factors affecting processing and accumulation of particular species. Understanding these factors will help to improve syn-tasiRNA efficiency and better exploit their potential to act on several targets with high specificity.

## Experimental procedures

### Plasmids

TAS1c was used as a backbone to produce syn-tasiRNA as previously described [28,29]. The synthetic tasiRNA constructs targeting the PPV genome were made using a 150 bp fragment including 2 nt from TAS1c, the miR173 target site (22 nt), and either the antisense strand of the CP region (9242–9367) (syn-tasiR-CP) or the 3’NCR region (9529–9654) (syn-tasiR-3NCR) of PPV. Gateway recombination sites were also added to the 5´ ends of the primers.

Primers for syn-tasiR-CP:

syn-tasiR-CP-F: 5´GGGGACAAGTTTGTACAAAAAAGCAGGCTTTGTGATTTTTCTCTACAAGCG

AATCAAAGGCATATCTGGCGAGG 3´

syn-tasiR-3NCR-R: 5´GGGGACCACTTTGTACAAGAAAGCTGGGTTATGGCACATTTCAGTAACGT 3´

Primers for syn-tasiR-3NCR:

syn-tasiR-3NCR-F: 5´GGGGACAAGTTTGTACAAAAAAGCAGGCTTTGTGATTTTTCTCTACAAGCGAAGAGAAAAGGATGCTAACAGGA 3´.

syn-tasiR-3NCR-R: 5´GGGGACCACTTTGTACAAGAAAGCTGGGTATCGCAACATGCACAACCTCC 3´.

The *Arabidopsis thaliana* MIR173 precursor (509nt) and MIR159 precursor (334nt) were amplified from Col-0 genomic DNA using the following primers, which included Gateway recombination sites:

MIR173-F: 5´GGGGACAAGTTTGTACAAAAAAGCAGGCTATAATTAGCAAGTAATAAGG3´ MIR173-R: 5´GGGGACCACTTTGTACAAGAAAGCTGGGTATCTGTTATACAACCAAATCC3´

MIR159-F: 5´GGGGACAAGTTTGTACAAAAAAGCAGGCTTTACAGTTTGCTTATGTCAGATCC3´

MIR159-R: 5´GGGGACCACTTTGTACAAGAAAGCTGGGTTGACCCGGGATGTAGAGCTCCCTTCAATCC3´

All the purified PCR fragments were introduced between the CaMV 35S promoter and the NOS terminator sequences into the pMDC32 binary vector by Gateway recombination and termed syn-tasiR-3NCR, syn-tasiR-CP, MIR173 and MIR159.

### Plant agroinfiltration

*Nicotiana benthamiana* plants were grown in a greenhouse maintained at 16 h light and 8 h dark photoperiod, temperature range 19–23°C. Plants with 5–6 true leaves were used for agroinfiltration as described [51]. Culture concentration was adjusted to OD_600_ = 1 for each construct, when two constructs were agroinfilrated a mixed 1:1 (v/v) culture was prepared.

### Virus inoculation and protein analysis

Two or three agroinfiltrated *N*. *benthamiana* leaves were dusted with carborundum and inoculated with 150 ng or 450 ng of purified GFP-tagged PPV [52]. Virus accumulation was assessed by Western blot analysis as described [53]. Ponceau red staining was used to confirm equivalent protein loading.

### GFP fluorescence imaging

Infected *N*. *benthamiana* leaves were screened for GFP expression with a MZ FLII (Leica Microsystems) fluorescence stereomicroscope, using excitation and barrier filters of 480/40 nm and 510 nm, respectively. Images were collected with an Olympus DP70 digital camera with DP controller and DP manager software.

### RNA isolation, Northern blot and Deep sequencing analysis

Small RNAs were purified from agroinfiltrated *N*. *benthamiana* leaves and subjected to Northern blot analysis as previously described [54].

RNA samples from twelve leaves (2 leaves from each plant) agroinfiltrated with two independent clones of syn-tasiR-3NCR and two independent clones of syn-tasiR-CP, either in the presence or absence of miR173 precursor, were pooled. Small RNA libraries were prepared and subjected to deep sequencing by the Beijing Genomics Institute (BGI-Shenzhen, Shenzhen, China). Briefly, between 15–30 nucleotides (nt) RNAs were purified and ligated to the Illumina 5´ and 3´ adaptors, and further converted into single-stranded cDNA and then amplified by PCR. Purified PCR products were sequenced by an Illumina HiSeq 2000 Genome Analyzer.

The high throughput sequencing data are freely available at GEO database http://www.ncbi.nlm.nih.gov/geo/query/acc.cgi?acc=GSE69348 (accession number GSE69348).

### Mapping sequence reads to reference sequences

Clean reads were obtained after adapter trimming and removing low quality reads, adaptor null reads, insert null reads, rRNA and tRNA and reads with polyA tail using the software of The UEA small RNA Workbench (http://srna-workbench.cmp.uea.ac.uk/tools/) [55]. Clean reads were mapped to reference using BWA [56] without mismatches. All identical sequences were counted and merged as unique sequences. The following analysis of read counts, as charts data, were performed by in-house-developed PHP and R scripts.

## Supporting Information

S1 Table Small RNA species induced by miR173 expression. Total small RNA species and 21nt, 22nt and 24nt species matching different sequences are given in columns. The overall number, the induced and its ratio are also detailed.
(DOCX)

## References

[1] Baulcombe D. (2005). RNA silencing. Trends Biochem. Sci. 30, 290–293. 15950871 10.1016/j.tibs.2005.04.012

[2] Axtell M.J. (2013). Classification and comparison of small RNAs from plants. Annu. Rev. Plant Biol. 64, 137–59. 10.1146/annurev-arplant-050312-120043 23330790

[3] Yoshikawa M. (2013). Biogenesis of trans-acting siRNAs, endogenous secondary siRNAs in plants. Genes Genet. Syst. 88, 77–84. 23832299 10.1266/ggs.88.77

[4] Wei K.F., Wu L.J., Chen J., Chen Y.F. and Xie D.X. (2012). Structural evolution and functional diversification analyses of argonaute protein. J. Cell. Biochem. 113, 2576–2585. 10.1002/jcb.24133 22415963

[5] Voinnet O. (2009). Origin, biogenesis, and activity of plant microRNAs. Cell 136, 669–687. 10.1016/j.cell.2009.01.046 19239888

[6] Eamens A., Wang M.B., Smith N.A. and Waterhouse P.M. (2008). RNA silencing in plants: Yesterday, today, and tomorrow. Plant Physiol. 147, 456–468. 10.1104/pp.108.117275 18524877 PMC2409047

[7] Schwab R. and Voinnet O. (2010). RNA silencing amplification in plants: Size matters. Proc. Natl. Acad. Sci. U.S.A. 107, 14945–6. 10.1073/pnas.1009416107 20709960 PMC2930571

[8] Baulcombe D.C. (2007). Amplified silencing. Science 315, 199–200. 17218517 10.1126/science.1138030

[9] Voinnet O. (2008). Use, tolerance and avoidance of amplified RNA silencing by plants. Trends Plant Sci. 13, 317–328. 10.1016/j.tplants.2008.05.004 18565786

[10] Wang X.B., Jovel J., Udomporn P., Wang Y., Wu Q., Li W-X., et al (2011). The 21-nucleotide, but not 22-nucleotide, viral secondary small interfering RNAs direct potent antiviral defense by two cooperative Argonautes in *Arabidopsis thaliana* . Plant Cell 23, 1625–1638. 10.1105/tpc.110.082305 21467580 PMC3101545

[11] Herr A.J., Molnar A., Jones A. and Baulcombe D.C. (2006). Defective RNA processing enhances RNA silencing and influences flowering of Arabidopsis. Proc. Natl. Acad. Sci. U.S.A. 103, 10994–15001. 10.1073/pnas.0606536103 PMC1581427 17008405

[12] Axtell M.J., Jan C., Rajagopalan R. and Bartel D.P. (2006). A two-hit trigger for siRNA biogenesis in plants. Cell 127, 565–577. 17081978 10.1016/j.cell.2006.09.032

[13] Manavella P.A., Koenig D. and Weigel D. (2012). Plant secondary siRNA production determined by microRNA-duplex structure. Proc. Natl. Acad. Sci. U.S.A. 109, 2461–2466. 10.1073/pnas.1200169109 22308502 PMC3289316

[14] Montgomery T.A., Howell M.D., Cuperus J.T., Li D., Hansen J.E., Alexander A.L., et al (2008). Specificity of ARGONAUTE7-miR390 interaction and dual functionality in TAS3 trans-acting siRNA formation. Cell 133, 128–141. 10.1016/j.cell.2008.02.033 18342362

[15] Chen H.-M., Chen L.-T., Patel K., Li Y.-H., Baulcombe D.C. and Wu S.-H. (2010). 22-nucleotide RNAs trigger secondary siRNA biogenesis in plants. Proc. Natl. Acad. Sci. U.S.A. 107, 15269–15274. 10.1073/pnas.1001738107 20643946 PMC2930544

[16] Cuperus J.T., Carbonell A., Fahlgren N., García-Ruiz H., Burke R.T., Takeda A., et al (2010). Unique functionality of 22-nt miRNAs in triggering RDR6-dependent siRNA biogenesis from target transcripts in *Arabidopsis* . Nat. Struct. Mol. Biol. 17, 997–1003. 10.1038/nsmb.1866 20562854 PMC2916640

[17] Rajeswaran R., Aregger M., Zvereva A.S., Borah B.K., Gubaeva E.G. and Pooggin M.M. (2012). Sequencing of RDR6-dependent double-stranded RNAs reveals novel features of plant siRNA biogenesis. Nucleic Acids Res. 40, 6241–54. 10.1093/nar/gks242 22434877 PMC3401431

[18] Fei Q., Xia R. and Meyers B.C. (2013). Phased, secondary, small interfering RNAs in posttranscriptional regulatory networks. Plant Cell 25, 2400–15. 10.1105/tpc.113.114652 23881411 PMC3753373

[19] Chitwood D.H., Guo M., Nogueira F.T. and Timmermans M.C. (2007). Establishing leaf polarity: the role of small RNAs and positional signals in the shoot apex. Development 134, 813–23. 17251271 10.1242/dev.000497

[20] Fahlgren N., Montgomery T.A., Howell M.D., Allen E., Dvorak S.K., Alexander A.L., et al (2006). Regulation of *AUXIN RESPONSE FACTOR3* by *TAS3* ta-siRNA affects developmental timing and patterning in *Arabidopsis* . Curr. Biol. 16, 939–944. 16682356 10.1016/j.cub.2006.03.065

[21] Howell M.D., Fahlgren N., Chapman E.J., Cumbie J.S., Sullivan C.M., Givan S.A., et al (2007). Genome-wide analysis of the RNA-DEPENDENT RNA POLYMERASE6/DICER-LIKE4 pathway in *Arabidopsis* reveals dependency on miRNA- and tasiRNA-directed targeting. Plant Cell 19, 926–942. 17400893 10.1105/tpc.107.050062 PMC1867363

[22] Chen H.M., Li Y.H. and Wu S.H. (2007). Bioinformatic prediction and experimental validation of a microRNA-directed tandem trans-acting siRNA cascade in Arabidopsis. Proc. Natl. Acad. Sci. U.S.A. 104, 3318–3323. 17360645 10.1073/pnas.0611119104 PMC1805617

[23] Li F., Pignatta D. Bendix C., Brunkard J.O., Cohn M.M., Tung J. et al (2012). MicroRNA regulation of plant innate immune receptors. Proc. Natl. Acad. Sci. U.S.A. 109, 1790–1795. 10.1073/pnas.1118282109 22307647 PMC3277104

[24] Shivaprasad P.V., Chen H.M., Patel K., Bond D.M., Santos B.A. and Baulcombe D.C. (2012). A microRNA superfamily regulates nucleotide binding site-leucine-rich repeats and other mRNAs. Plant Cell 24, 859–874. 10.1105/tpc.111.095380 22408077 PMC3336131

[25] Zhai J., Jeong D-H., De Paoli E., Park S., Rosen B.D., Li Y. et al (2011). MicroRNAs as master regulators of the plant NB-LRR defense gene family via the production of phased, trans-acting siRNAs. Genes Dev. 25, 2540–53. 10.1101/gad.177527.111 22156213 PMC3243063

[26] Boccara M., Sarazin A., Thiébeauld O., Jay F., Voinnet O., Navarro L., et al (2014). The arabidopsis *miR472-RDR6* silencing pathway modulates PAMP- and effector-triggered immunity through the post-transcriptional control of disease resistance genes. PLoS Pathog. 10, e1003883 10.1371/journal.ppat.1003883 24453975 PMC3894208

[27] Cuperus J.T., Fahlgren N. and Carrington J.C. (2011). Evolution and functional diversification of *MIRNA* genes. Plant Cell 23, 431–442. 10.1105/tpc.110.082784 21317375 PMC3077775

[28] Gutierrez-Nava M.D., Aukerman M.J., Sakai H., Tingey S.V. and Williams R.W. (2008). Artificial trans-acting siRNAs confer consistent and effective gene silencing. Plant Physiol. 147, 543–551. 10.1104/pp.108.118307 18441221 PMC2409013

[29] Montgomery T.A., Yoo S.J., Fahlgren N., Gilbert S.D., Howell M.D., Sullivan C.M. et al (2008). AGO1-miR173 complex initiates phased siRNA formation in plants. Proc. Natl. Acad. Sci. U.S.A. 105, 20055–20062. 10.1073/pnas.0810241105 19066226 PMC2598728

[30] Felippes F.F. and Weigel D. (2009). Triggering the formation of tasiRNAs in Arabidopsis thaliana: the role of microRNA miR173. EMBO Rep. 10, 264–270. 10.1038/embor.2008.247 19180117 PMC2658565

[31] Carbonell A., Takeda A., Fahlgren N., Johnson S.C., Cuperus J.T. and Carrington J.C. (2014). New generation of artificial microRNA and synthetic trans-acting small interfering RNA vectors for efficient gene silencing in Arabidopsis. Plant Physiol. 165, 15–29. 10.1104/pp.113.234989 24647477 PMC4012576

[32] de Felippes F.F., Wang J.W. and Weigel D. (2012). MIGS: miRNA-induced gene silencing. Plant J. 70, 541–7. 10.1111/j.1365-313X.2011.04896.x 22211571

[33] Qu J., Ye J. and Fang R. (2012). Artificial microRNAs for plant virus resistance. Methods Mol. Biol. 894, 209–22. 10.1007/978-1-61779-882-5_14 22678582

[34] García J.A. and Simón-Mateo C. (2006). A micropunch against plant viruses. Nat. Biotechnol. 24, 1358–1359. 17093480 10.1038/nbt1106-1358

[35] Tiwari M., Sharma D. and Trivedi P.K. (2014). Artificial microRNA mediated gene silencing in plants: progress and perspectives. Plant Mol. Biol. 86, 1–18. 10.1007/s11103-014-0224-7 25022825

[36] García J.A., Glasa M., Cambra M. and Candresse T. (2014). *Plum pox virus* and sharka: a model potyvirus and a major disease. Mol. Plant Pathol. 15, 226–241. 10.1111/mpp.12083 24102673 PMC6638681

[37] Valli A., Oliveros J.C., Molnar A., Baulcombe D. and García J.A. (2011). The specific binding to 21-nt double-stranded RNAs is crucial for the anti-silencing activity of *Cucumber vein yellowing virus* P1b and perturbs endogenous small RNA populations. RNA 17, 1148–1158. 10.1261/rna.2510611 21531919 PMC3096046

[38] Simón-Mateo C. and García J.A. (2011). Antiviral strategies in plants based on RNA silencing. Biochim. Biophys. Acta 1809, 722–731. 10.1016/j.bbagrm.2011.05.011 21652000

[39] Smith N.A., Singh S.P., Wang M.B., Stoutjesdijk P.A., Green A.G. and Waterhouse P.M. (2000). Total silencing by intron-spliced hairpin RNAs. Nature 407, 319–320. 11014180 10.1038/35030305

[40] Béclin C., Boutet S., Waterhouse P. and Vaucheret H. (2002). A branched pathway for transgene-induced RNA silencing in plants. Curr. Biol. 12, 684–688. 11967158 10.1016/s0960-9822(02)00792-3

[41] Niu Q.-W., Lin S.-S., Reyes J.L., Chen K.-C., Wu H.-W., Yeh S.-D., et al (2006). Expression of artificial microRNAs in transgenic *Arabidopsis thaliana* confers virus resistance. Nat. Biotechnol. 24, 1420–1428. 17057702 10.1038/nbt1255

[42] Duan C.G., Wang C.H., Fang R.X. and Guo H.S. (2008). Artificial MicroRNAs highly accessible to targets confer efficient virus resistance in plants. J. Virol. 82, 11084–11095. 10.1128/JVI.01377-08 18768978 PMC2573272

[43] Carbonell A., Fahlgren N., Garcia-Ruiz H., Gilbert K.B., Montgomery T.A., Nguyen T., et al (2012). Functional analysis of three *Arabidopsis* ARGONAUTES using slicer-defective mutants. Plant Cell 24, 3613–29. 10.1105/tpc.112.099945 23023169 PMC3480291

[44] Allen E., Xie Z.X., Gustafson A.M. and Carrington J.C. (2005). microRNA-directed phasing during *trans*-acting siRNA biogenesis in plants. Cell 121, 207–221. 15851028 10.1016/j.cell.2005.04.004

[45] Gasciolli V, Mallory AC, Bartel DP, Vaucheret H (2005) Partially redundant functions of Arabidopsis DICER-like enzymes and a role for DCL4 in producing trans-acting siRNAs. Curr Biol 15: 1494–1500. 16040244 10.1016/j.cub.2005.07.024

[46] Henderson IR, Zhang X, Lu C, Johnson L, Meyers BC, Green P.J., et al (2006) Dissecting Arabidopsis thaliana DICER function in small RNA processing, gene silencing and DNA methylation patterning. Nat Genet 38: 721–725. 16699516 10.1038/ng1804

[47] Toedling J., Servant N., Ciaudo C., Farinelli L., Voinnet O., Heard E. et al (2012). Deep-sequencing protocols influence the results obtained in small-RNA sequencing. PLoS One 7, e32724 10.1371/journal.pone.0032724 22384282 PMC3287988

[48] Mi S, Cai T, Hu Y, Chen Y, Hodges E, Ni F., et al (2008) Sorting of small RNAs into Arabidopsis argonaute complexes Is directed by the 5' terminal nucleotide. Cell 133: 116–127. 10.1016/j.cell.2008.02.034 18342361 PMC2981139

[49] Khvorova A, Reynolds A, and Jayasena SD (2003) Functional siRNAs and miRNAs exhibit strand bias. Cell 115: 209–216. 14567918 10.1016/s0092-8674(03)00801-8

[50] Schwarz DS, Hutvagner G, Du T, Xu Z, Aronin N, and Zamore P.D. (2003) Asymmetry in the assembly of the RNAi enzyme complex. Cell 115: 199–208. 14567917 10.1016/s0092-8674(03)00759-1

[51] Valli A., Martín-Hernández A.M., López-Moya J.J. and García J.A. (2006). RNA silencing suppression by a second copy of the P1 serine protease of *Cucumber vein yellowing ipomovirus* (CVYV), a member of the family *Potyviridae* that lacks the cysteine protease HCPro. J. Virol. 80, 10055–10063. 17005683 10.1128/JVI.00985-06 PMC1617295

[52] Fernández-Fernández M.R., Mouriño M., Rivera J., Rodríguez F., Plana-Durán J. and García J.A. (2001). Protection of rabbits against rabbit hemorrhagic disease virus by immunization with the VP60 protein expressed in plants with a potyvirus-based vector. Virology 280, 283–291. 11162842 10.1006/viro.2000.0762

[53] Carbonell A., Maliogka V.I., Pérez J.J., Salvador B., San León D., García J.A. et al (2013). Diverse amino acid changes at specific positions in the N-terminal region of the coat protein allow *Plum pox virus* to adapt to new hosts. Mol. Plant Microbe Interact. 26, 1211–24. 10.1094/MPMI-04-13-0093-R 23745677

[54] Simón-Mateo C. and García J.A. (2006). MicroRNA-guided processing impairs *Plum pox virus* replication, but the virus readily evolves to escape this silencing mechanism. J. Virol. 80, 2429–2436. 16474149 10.1128/JVI.80.5.2429-2436.2006 PMC1395392

[55] Stocks M.B., Moxon S., Mapleson D., Woolfenden H.C., Mohorianu I., Folkes L., et al (2012). The UEA sRNA workbench: a suite of tools for analysing and visualizing next generation sequencing microRNA and small RNA datasets. Bioinformatics 28, 2059–61. 10.1093/bioinformatics/bts311 22628521 PMC3400958

[56] Li H. and Durbin R. (2010). Fast and accurate long-read alignment with Burrows-Wheeler transform. Bioinformatics 26, 589–95. 10.1093/bioinformatics/btp698 20080505 PMC2828108

