# Supplementary material for: Assorted Processing of Synthetic Trans-Acting siRNAs and Its Activity in Antiviral Resistance
Source: PLoS One. 2015 Jul 6;10(7):e0132281. doi: 10.1371/journal.pone.0132281 (PMC4492489; doi:10.1371/journal.pone.0132281)
Supplement: S1 Table — Total small RNA species and 21nt, 22nt and 24nt species matching different sequences are given in columns. The overall number, the induced and its ratio are also detailed. (DOCX) [file pone.0132281.s001.docx]

Supplementary Table 1. Small RNA species induced by miR173 expression

| miR173-induced species | Total | | | 21 nt | | | 22 nt | | | 24 nt | | |
| --- | --- | --- | --- | --- | --- | --- | --- | --- | --- | --- | --- | --- |
|  | Induced | Total | Ratio (%) | Induced | Total | Ratio (%) | Induced | Total | Ratio (%) | Induced | Total | Ratio (%) |
| *N. benthamiana* | 1 | 2502221 | 0,00 | 0 | 156894 | 0 | 0 | 153255 | 0 | 0 | 1598996 | 0 |
| *Agrobacterium* | 0 | 38118 | 0 | 0 | 3400 | 0 | 0 | 3192 | 0 | 0 | 3021 | 0 |
| Others | 7 | 243619 | 0,00 | 0 | 32941 | 0 | 1 | 32207 | 0,00 | 3 | 53125 | 0,01 |
| pMDC32-common | 9 | 164932 | 0,01 | 7 | 34698 | 0,02 | 1 | 43168 | 0,00 | 0 | 44231 | 0 |
| CP | 34 | 3768 | 0,90 | 9 | 832 | 1,08 | 1 | 677 | 0,15 | 3 | 905 | 0,33 |
| 3NCR | 22 | 3626 | 0,61 | 13 | 938 | 1,39 | 2 | 743 | 0,27 | 0 | 745 | 0 |

Total: Number of small RNA reads.
